# Steroid hormone responses to three exercise modalities assessed by liquid chromatography tandem mass spectrometry in a randomized crossover trial

**DOI:** 10.1038/s41598-026-58281-9

**Published:** 2026-06-29

**Authors:** D. McCullough, P. Ferentinos, N. Z. M. Homer, A. Basiukajć, D. R. Woods, R. M. Gifford, T. Ispoglou

**Affiliations:** 1 Carnegie School of Sport, Leeds Beckett University, Leeds, Yorkshire UK; 2 Mass Spectrometry Core, Centre for Cardiovascular Sciences, University of Edinburgh, Edinburgh, UK; 3 Academic Department of Military Medicine, Royal Centre of Defence Medicine, Birmingham, UK

**Keywords:** Moderate-intensity continuous exercise, High-intensity intermittent exercise, Hormones, Resistance exercise, Steroids, Biochemistry, Endocrinology, Physiology

## Abstract

Exercise disrupts hormonal balance, driving physiological adaptations. However, inconsistent findings exist due to methodological limitations. To address this, this study simultaneously profiled 16 steroid hormones via LC-MS/MS in response to three distinct exercise modalities – moderate intensity continuous exercise (MICE), resistance exercise (RE), and High-intensity Intermittent Exercise (HIIE) – using a randomized crossover design. Ten healthy adult males completed HIIE (> 80% V̇O_2max_), MICE (< 65% V̇O_2max_) and whole-body RE (3 sets×10 reps at 70% 1RM with 90 s rest) in a randomized sequence. Blood samples were collected pre-, 10 min post-, and 2 h post-exercise, and analyzed for glucose, lactate, and steroid hormones. DHEA and androstenedione increased post-exercise following MICE, HIIE (both *P* < 0.01), and RE (*P* < 0.05), but declined below rest by 2 h. Estrone increased 2 h post-RE vs. post-exercise (*P* < 0.01), and 2 h post-HIIE vs. rest (*P* < 0.01). Only HIIE elevated cortisol (*P* < 0.001) and cortisone (*P* < 0.01) post-exercise, while cortisol fell below rest at 2 h across all modalities (*P* < 0.001). HIIE also raised corticosterone (*P* < 0.001), exceeding post-MICE (*P* < 0.01) which showed sub-resting corticosterone at 2 h post-exercise (*P* < 0.01). All exercise modalities increased aldosterone (*P* < 0.01), with greater responses in MICE and HIIE than RE. At 2 h, all protocols elevated the testosterone: cortisol ratio (*P* < 0.05). RE and HIIE altered sex hormone ratios, decreasing testosterone:17b-estradiol and androgens: estrogens, and increasing estrogens: progesterone (*P* < 0.05). These exploratory findings demonstrate mode-specific acute hormonal responses in young males. Future studies should explore circulating hormonal tissue interactions to clarify potential functional adaptations.

**Supplementary Information:**

The online version contains supplementary material available at 10.1038/s41598-026-58281-9.

## Introduction

Exercise rapidly increases metabolism by up to 100-fold^1^, requiring sophisticated control mechanisms to adjust substrate utilization, to meet energy demands and restore homeostasis^1^. Acute alterations in substrates and metabolites, coupled with mechanical stresses on the body, result in specific adaptations through chronic repetition^2^. These adaptations enhance exercise performance and are associated with broader health benefits^3^. Understanding these mechanisms is essential for developing physiological insight and informing training approaches.

Exercise influences all major steroid hormone classes – androgens, estrogens, progestins, and corticosteroids – via changes in secretion, clearance, degradation, and plasma volume, enhancing hormone-receptor interactions^4–6^. Hormones regulate metabolism and may also play a role in exercise-induced adaptations, energy utilization and recovery^7^. For example, whole-body resistance exercise (RE) triggers peak anabolic responses at 15 min^8^ and therefore have been studied extensively as potential mediators of RE-induced muscle hypertrophy however, a large body of evidence suggests that transient post-exercise rises in androgen concentrations are unrelated to muscle hypertrophy^9–11^. Intriguingly, endurance exercise has also been shown to increase androgen levels in males, with 5α-dihydrotestosterone (DHT) and testosterone peaking immediately until 15–30 min post exercise respectively, depending on intensity^12–14^. Therefore, elevated androgens might be more indicative of motivation or neuromuscular performance^15,16^ rather than long-term muscle growth. Furthermore, in males, high intensity endurance exercise elicits immediate post-exercise rises in 17b-estradiol and cortisol^12^, with cortisol remaining elevated up to 30 min of recovery, whereas lower intensities do not^17,18^. These intensity-dependent responses may provide insight into exercise prescription and recovery processes^19,20^. Moreover, tailoring training according to hormonal responses may offer insight into age-related endocrine, musculoskeletal and metabolic changes, particularly in older adults at risk of sarcopenia^21^.

Accurately assessing hormonal responses to exercise requires careful methodological consideration. Commonly used enzyme-linked immunosorbent assays (ELISAs) lack the specificity, sensitivity, and accuracy^22,23^ of liquid chromatography with tandem mass spectrometry (LC-MS/MS), which enhances the detection of exercise-induced hormonal changes^24^ and enables profiling of multiple steroid hormones simultaneously. Consequently, reliance on ELISAs in past studies may have led to inaccuracies and inconsistent findings. Despite generally acceptable assay variability, ELISA-derived measures can show substantial intra-individual variability, which may constrain detection of subtle exercise-induced hormonal changes^14,25^. In contrast, LC-MS/MS demonstrates greater analytical precision and reliability for steroid hormone measurement, enabling more robust detection of small, physiologically meaningful differences^26^. Furthermore, LC-MS/MS limits of quantitation for testosterone and estrogens can be substantially lower than immunoassays^24^. Additionally, hormone levels are influenced by factors such as genetics, age, sex, circadian rhythms, body composition, stress, nutritional status, and health status^27,28^, which complicates parallel protocol comparisons between different exercise modes.

The overall aim of the study is to explore the hormonal responses to exercise, with an emphasis on acute, exploratory comparisons across exercise modalities. This study utilizes a randomized crossover design to compare the hormonal responses of three distinct exercise modalities (a) moderate-intensity continuous exercise (MICE); (b) RE and (c) high-intensity interval exercise (HIIE) in recreationally trained adult males.

We specifically investigate how differing exercise intensities and stressors influence key bioactive steroid hormones, including DHT, testosterone, cortisol, and 17b-estradiol, 10 min post-exercise and 2 h post-exercise using LC-MS/MS to overcome limitations of previous immunoassay-based studies. We further examine how these bioactive hormones are regulated by upstream corticosteroids, androgens, estrogens, and progestins.

## Materials and methods

### Participant screening and study protocol

All participants provided written informed consent, and all procedures were approved by the Leeds Beckett University Research Ethics Committee (REC number: 78966). All procedures followed the CONSORT guidelines for reporting randomised trials (Table S1, Fig S1)^29^. In a randomized crossover design, male adults from Leeds Yorkshire area, were invited between February 2019 and December 2019 to complete three exercise protocols under fasting conditions: (1) HIIE, (2) MICE, and (3) RE). Participants were included if they were male, aged 18–35 years, with a BMI of 18.5–29.9 kg/m^2^ and recreationally active, defined according to ACSM guidelines^30^ as engaging in structured physical activity for at least 30 min at moderate intensity on ≥ 3 days per week for a minimum of three months prior to enrolment. Participants were excluded if they were smokers, took dietary supplements, consumed alcohol above the weekly UK recommendations, had a history of metabolic diseases, or took lipid, blood pressure or blood glucose-lowering medication. Following informed consent, eligible participants were confirmed to meet the inclusion criteria, by providing a fasted finger prick blood test (Cholestech LDX^®^, Alere Ltd., Waltham, US), blood pressure, waist measurements, and by completing the International Physical Activity Questionnaire (IPAQ)^31^ and QRISK 3–2017 form^32^. Participants were next randomly allocated to groups using a computer-generated sequence, (Research Randomizer, https://www.randomizer.org/)^33^ using a simple randomization procedure, and the same investigator enrolled the participants. Following completion of the IPAQ form, participants’ weekly total physical activity levels were calculated and expressed in metabolic equivalents (MET)-minutes per week (MET.min.wk^− 1^) according to the questionnaire’s scoring guidelines^31^.

### Exercise intensity selection

Participants performed an incremental ramp cardiopulmonary exercise test (CPET) on an electromagnetically braked cycle ergometer (Lode, Corival Rehab, Netherlands). The work rates were calculated individually, using the Hansen/Wasserman equation, to ensure that participants terminated the test in 8–12 min, which ensures a more reliable assessment of V̇O_2max_^34^. Oxygen consumption (V̇O_2_) and carbon dioxide production (V̇CO_2_) production were measured breath-by-breath using an on-line gas analysis system (Metalyzer 3B, Cortex Biophysik GmbH, Leipzig, Germany). Prior to testing, the gas analyser was calibrated according to the manufacturer’s instructions. During the CPET an electrocardiogram trace (Custo Cardio 130, Custo med GmbH, Ottobrunn, Germany) was recorded continuously. Heart rate, blood pressure, rating of perceived exertion (RPE) and peripheral capillary oxygen saturation (SpO_2_) were recorded every 2 min. Peak exertion was determined using the following criteria: (1) RER *≥* 1.10, (2) identification of a plateau (*≤* 150mL increase) in V̇O_2_ despite an increase in workload, and (3) HR in excess of 90% of the age predicted maximum (220-age)^35^. The test was terminated at the participant’s request (volitional exhaustion) or when the participant was unable to maintain the cadence above 50 rpm.

### Muscular strength assessment and protocol familiarization

On a separate day, participants were familiarized with the RE and HIIE protocols and performed a one repetition maximal (1RM) strength test in each exercise according to procedures previously described^36^. Following a 5 min general standardised warm-up on a cycle ergometer, participants performed a specific warm-up with a light resistance that allowed 5–10 repetitions in each exercise. Following a 1 min rest, resistance was increased by 10%-20% to allow the participants to complete 3–5 repetitions. After 2 min of rest, the resistance was increased by 10%-20%. Following 2–4 min rest, resistance was increased by 10%-20% to allow the participant to perform one repetition. If the attempt was successful, the previous step was repeated. If the participant failed, a 2–4 min rest period was provided, and the load decreased by 5%-10% to attempt to complete one repetition.

### Experimental protocol

Participants arrived at the laboratory between 07.00 and 10.00 following an overnight fast, with a minimum of seven days between every exercise session, and the time of day was repeated with each participant to minimise diurnal variation in hormones^37^. Participants were instructed to abstain from exercise and alcohol for 24 h and caffeine for 8 h prior to each visit, maintain regular sleep patterns, and replicate their evening meal before each trial using a 24 h food diary. Participants were also instructed to consume ~ 250 mL of water 1 h before laboratory arrival. These procedures were implemented to minimise variability in hormonal and metabolic responses between trials. During each experimental day, participants were allowed to drink water ad libitum in order to avoid significant fluid loss leading to exercise induced haemoconcentration^38^. With the participant resting in the supine position, a trained phlebotomist inserted a cannula in a suitable vein in the antecubital fossa, to allow collection of blood samples into plasma EDTA vacutainers prior to exercise, 10 min post-exercise (hereafter referred to as ‘post-exercise’) and 2 h post-exercise. Resting blood sample was collected immediately following cannulation and no complications (repeated cannulation) with blood sampling occurred. Samples were immediately stored on ice until centrifugation at 3,500 x g at 4 °C for 10 min and plasma was stored at -80 °C until analysis. Participants’ heart rate (Polar Electro, UK Ltd) was recorded throughout, and pre- and post-exercise lactate levels and RPE were recorded.

### Blood pressure, glucose and lactate

At rest, immediately post- and 2 h post-exercise, systolic and diastolic blood pressure were collected by an automatic blood pressure sphygmomanometer (Suntech Tango, M2, USA). Blood glucose and lactate were determined via capillary blood samples and analysed immediately using a biochemistry analyser (YSI 2900D, YSI Inc./Xylem Inc., Ohio, USA).

### High intensity intermittent exercise

The HIIE protocol utilized in this study, represents an acute bout conceptually aligned with high-intensity interval training and is based on the previous literature demonstrating improvements in endothelial function and cardiorespiratory fitness in heart failure, hypertension, obesity and metabolic syndrome patients^39–42^. After 5 min of warm- up at 45% of V̇O_2max_ (55% of HR_max_), the participants completed 4 sets of 4 min intervals at 80% of V̇O_2max_ (85–95% of HR_max_), interspersed with 3 min of active recovery at 45% of V̇O_2max_ (55% of HR_max_) on a cycle ergometer (Lode Corival Rehab, Netherlands). Intensity for the different sections of the protocol (warm-up, high intensity, active recovery) was determined based on the power-HR plot of the CPET for each participant.

### Moderate-intensity continuous exercise

During the MICE protocol, participants started with a 5 min warm up at 45% of V̇O_2max_ (50–55% of HR_max_) and then completed continuous exercise at 60–65% of V̇O_2max_ (70–80% HR_max_) (all performed on a cycle ergometer, Lode Corival Rehab, Lone holding, Netherlands). The duration of exercise varied for each participant and was determined from the CPET to equate the total work performed between MICE and HIIE protocols, as previously employed in the literature^43,44^.Total external work for HIIE was calculated as the sum of high-intensity and recovery intervals (Work = Power × Time; 4 × 240 s at 80% V̇O₂_max_ + 4 × 180 s at 45% V̇O₂_max_), and MICE duration was subsequently calculated as: MICE duration (s) = total HIIE work / power at 60–65% V̇O₂_max_, with total work expressed in kilojoules (kJ). This design has been suggested as an approach to minimize the risk of bias when comparing the efficacy of different aerobic protocols^45^.

### Resistance exercise

The RE protocol consisted of a whole-body resistance exercise programme designed to trigger large anabolic hormonal responses^8^. After a 5 min warm-up on a cycle ergometer, at 45% of V̇O_2max,_ and dynamic stretching of all major muscle groups, participants performed a typical “hypertrophy” type workout, which is also associated with high metabolic responses, considering ACSM resistance training recommendations^46^. This involved three sets of 10 repetitions at 70% of 1RM of the following exercises, in order: seated leg press, chest press, leg extension, lateral pull down, upright row and shoulder press. Recovery between exercises was 1.5–2 min, and 3 min between exercises. Participants were instructed to perform repetitions through the full range of motion, in a controlled manner, and breathing appropriately. There was no pause between eccentric and concentric phase, with a minimum pause between attempts. Finally, most of the exercises were performed predominantly on weight machines to reduce technical difficulty.

### Liquid-chromatography mass spectrometry for targeted steroid analysis

Sixteen steroids were quantified in plasma using supported liquid extraction followed by LC–MS/MS, described by Denham et al.^47^ and further details can be found in supplementary data (Table S1–4). Briefly, plasma samples, calibration standards (16-point curve, 0.0025–500 ng), quality controls, and blanks (150 µL) were dispensed into 96-well plates. Isotopically labelled internal standards were added (20 µL), and samples were processed using an automated supported liquid extraction system (Biotage Extrahera™). Following extraction with dichloromethane/propan-2-ol (98:2), eluates were dried under nitrogen (40 °C), reconstituted in water/methanol (70:30), and analysed by LC–MS/MS. Chromatographic separation was performed using an I-Class UPLC (Waters, UK) with a Kinetex C18 column (150 × 2.1 mm, 2.6 μm) and ammonium fluoride-modified water/methanol mobile phase (0.3 mL·min⁻¹). Detection was performed using a QTRAP 6500 + mass spectrometer (AB Sciex, UK) operating in electrospray ionisation with polarity switching. Multiple reaction monitoring transitions were optimised for each steroid, with two transitions (quantifier and qualifier) used for identification. Data acquisition and integration were performed using Analyst^®^ 1.6.3 and MultiQuant 3.0.3 (Sciex). Analytical performance characteristics were derived from the validated method of Denham et al.^47^. Lower limits of quantification (LLOQ) ranged from 0.0125 to 5 ng·mL⁻¹ depending on analyte, with inter-assay precision of 2.4–19% and accuracy within − 12 to 11%. Recovery ranged ~ 90–120%, and isotopically labelled internal standards were used to account for matrix effects. Values below the LLOQ were imputed as LLOQ/3 (0.0125/3 ng·mL⁻¹) to minimize bias associated with zero substitution and enable statistical analysis. Only one sample measured below the LLOQ for 17b-estradiol and ten samples for 21-deoxycortisol and subsequently were analyzed. Sixty-one samples of 11-deoxycorticosterone were below the LLOQ and did not undergo statistical analysis.

### Statistical analysis

No a priori power calculation was performed due to the range of endocrine outcomes assessed. Sample size was based on comparable exercise endocrinology studies^14,47,48^, and hormonal responses should therefore be considered exploratory and hypothesis-generating. All data were explored for normal distribution using the Shapiro-Wilks test and visual inspection of normality plots. Parametric data are presented as mean ± SD. Non-normally distributed data were log or square root transformed prior to parametric analysis and raw data are presented or underwent non-parametric analysis and are presented as median (interquartile range (IQR)). To examine the effects of exercise conditions and timepoints, a 3 × 3 mixed ANOVA was carried out on cortisone, testosterone, androstenedione, cortisol, 11-deoxycortisol, 5a-DHT, 17b-estradiol, corticosterone, aldosterone, lactate and testosterone to cortisol ratio to detect significant main and interaction effects. Area under curve (AUC) was calculated using the trapezoid equation ($\:\frac{\left(x2-x1\right)\:x\:(y1+y2)}{2}$) to estimate hormone concentration–time exposure and presented as ng.h which underwent one way ANOVA analysis. Significant main effects or interactions identified by ANOVA were followed by post hoc comparisons. Remaining non-parametric data, were analyzed using Friedman tests with Wilcoxon signed-rank post hoc tests, and between-condition comparisons using Kruskal-Wallis and Mann-Whitney U tests. Effect sizes are reported as partial eta squared (ηp²) for parametric analyses and r for non-parametric tests. Where appropriate, mean differences and 95% confidence intervals are provided for key pairwise comparisons of parametric data. Holm–Bonferroni correction was applied for multiple comparisons (*P* < 0.05) and SPSS (version 29, IBM Chicago, USA) statistical software was used for analysis.

## Results

### The effect of exercise on body mass, cardiovascular and metabolic metrics

**Fig. 1:**
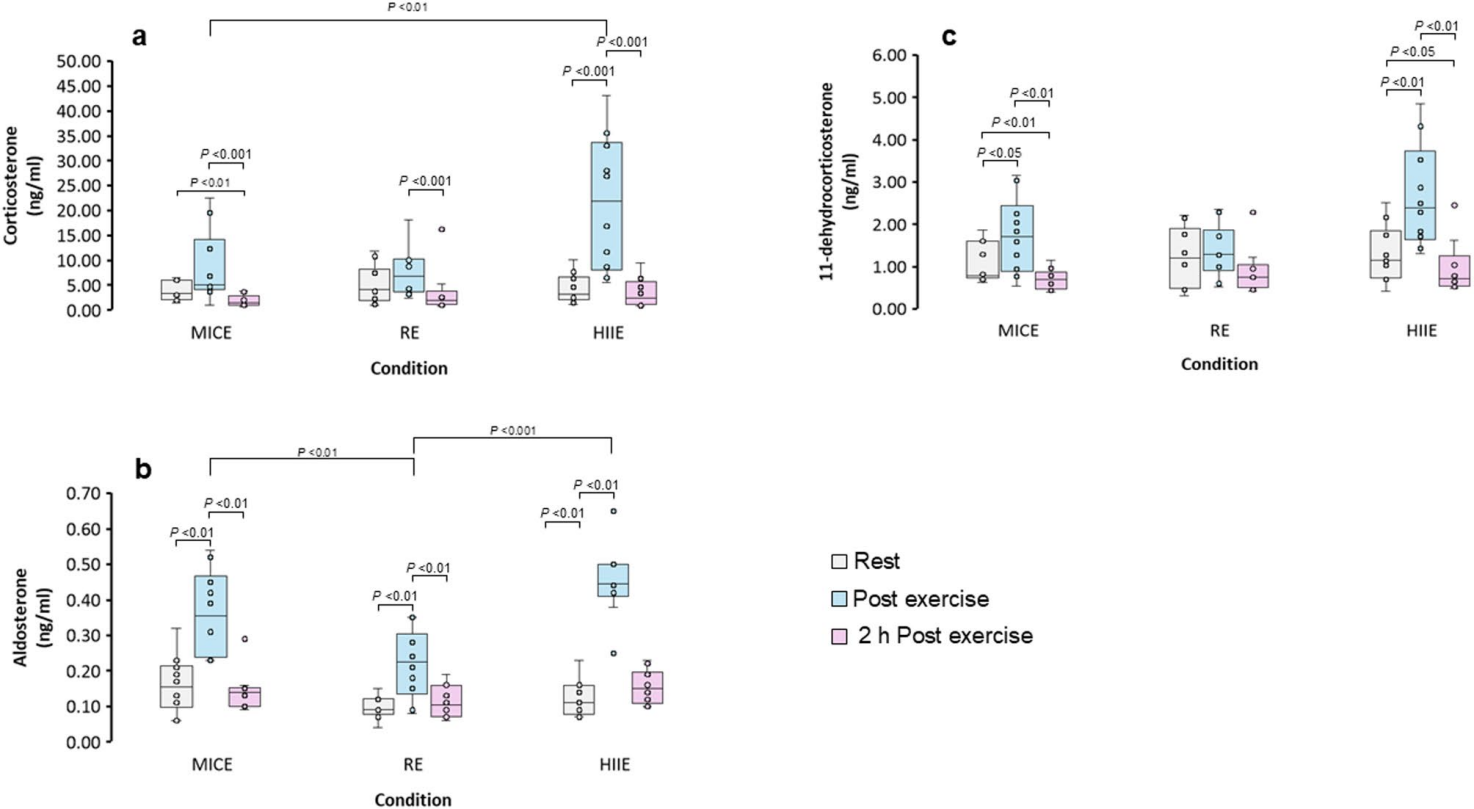
The effect of exercise on corticosteroids and mineralocorticoids via the progesterone pathway, (**a**) Corticosterone, (**b**) Aldosterone comparisons made by two-way mixed ANOVA. (**c**) 11-dehydrocorticosterone comparisons made by Wilcoxon signed rank and Mann Whitney U tests. Holm-Bonferroni post hoc corrections were applied to all comparisons (*n* = 10 per group). HIIE, High-intensity intermittent exercise; MICE, moderate-intensity continuous exercise; RE, resistance exercise.

**Fig. 2:**
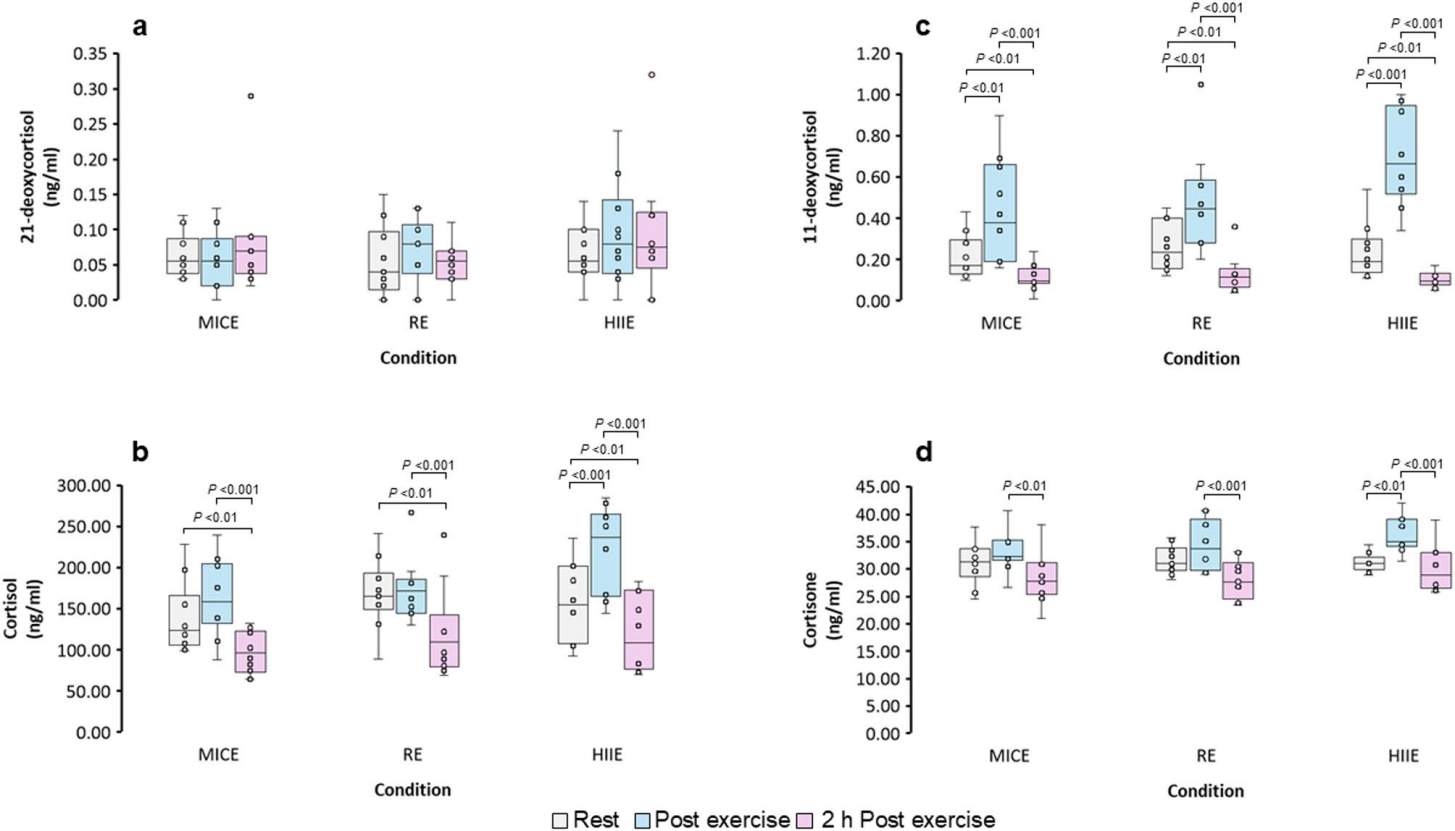
The effect of exercise on corticosteroids via the 17OH-progesterone pathway, (**a**) 21-deoxycortisol, comparisons made by Wilcoxon signed rank and Mann Whitney U tests. (**b**) Cortisol, (**c**) 11-dexoycortisol, c) Cortisone, comparisons made by two-way mixed ANOVA. Holm-Bonferroni post hoc corrections were applied to all comparisons (*n* = 10 per group). HIIE, High-intensity intermittent exercise; MICE, moderate-intensity continuous exercise; RE, resistance exercise.

**Fig. 3:**
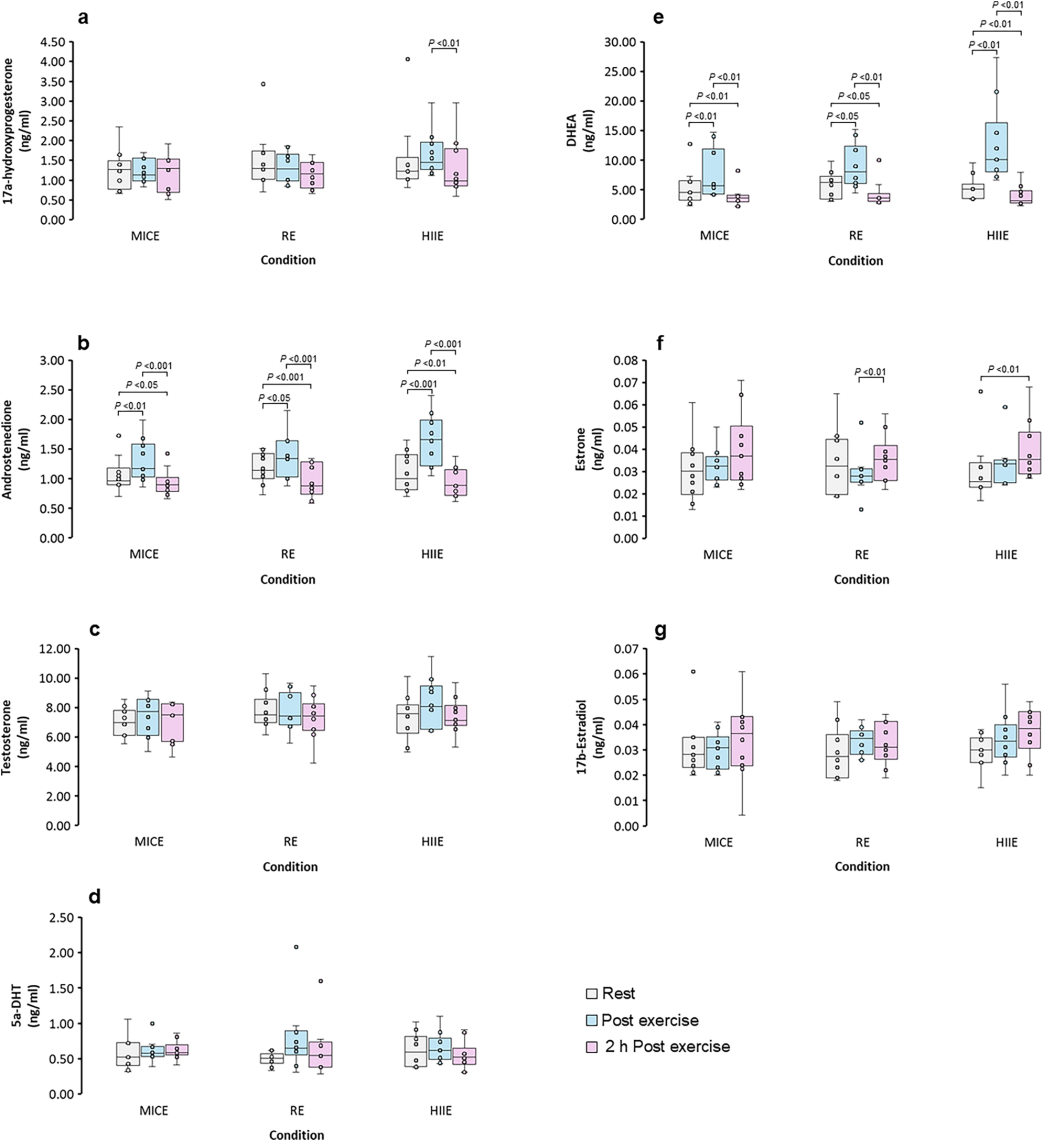
The effect of exercise on gonadal steroids, (**a**) 17a-hydroxyprogesterone, (**b**) Androstenedione, (**e**) DHEA, (**f**) Estrone, (**g**) 17b-Estradiol comparisons made by Wilcoxon signed rank and Mann Whitney U tests. (**c**) Testosterone, (**d**) 5a-DHT comparisons made by two-way mixed ANOVA. Holm-Bonferroni post hoc corrections were applied to all comparisons (*n* = 10 per group). 5a-DHT, 5-alpha-dihydrotestosterone; DHEA, Dehydroepiandrosterone; HIIE, High-intensity intermittent exercise; MICE, moderate-intensity continuous exercise; RE, resistance exercise.

Ten male (24.6 ± 2.1 years old) highly active participants with a BMI of 25.7 +/- 3.3 kg/m^2^ underwent a CPET and 1 RM testing (Table S2) and randomly completed the MICE, RE and HIIE protocols on 3 separate occasions at the same time of day. No harms or unintended events were reported in any of the exercise protocols. There was no difference (*P* = 0.23) in the mean mechanical work done between the HIIE (293.8 ± 29.4 kJ) and MICE 311.4 ± 34.2 kJ protocols. There were no differences (*P* > 0.05) in pre-exercise levels of body mass, blood pressure, resting heart rate, blood glucose, blood lactate (Table 3) or circulating steroid hormones (Figs. 1, 2 and 3) between exercise conditions.

All exercise modes resulted in decreases (*P* = 0.02) in body mass. MICE and HIIE resulted in significant (*P* < 0.01) increases in post exercise systolic blood pressure respectively and returned to resting levels at 2 h post exercise. However, only HIIE resulted in a significant (*P* < 0.01) increase in post exercise diastolic blood pressure. RE did not increase systolic blood pressure or diastolic blood pressure and was significantly (*P* < 0.01) lower post exercise compared to MICE and HIIE. All exercise modes increased (*P* < 0.01) post exercise heart rate and were not different to each other. MICE (13.90 +/- 1.85) resulted in significantly lower RPE compared with RE (15.80 +/- 1.69, *P* = 0.01) and HIIE (16.80 +/- 1.23, *P* < 0.001). All conditions increased (*P* < 0.001) post exercise blood lactate concentrations with a significant time effect (F(2, 54) = 351.46, *P* < 0.001, ηp² =0.93) and a condition × time interaction (F(4, 54) = 10.36, *P* < 0.001, ηp² =0.43). However, RE and HIIE post exercise blood lactate concentrations were significantly higher (*P* < 0.001) compared with MICE and only RE remained significantly (*P* < 0.01) elevated at 2 h post exercise compared with resting concentrations. Similarly, MICE (13.90 +/- 1.85) resulted in significantly lower RPE compared with RE (15.80 +/- 1.69, *P* = 0.01) and HIIE (16.80 +/- 1.23, *P* < 0.001). Blood glucose levels did not change with MICE but RE decreased blood glucose levels at post (*P* < 0.01, *r* = 0.89) and 2 h post exercise (*P* < 0.01, *r* = 0.79). HIIE elevated post exercise blood glucose concentrations compared with MICE (*P* < 0.05 *r* = 0.74) and RE (*P* < 0.05 *r* = 0.76) and were significantly (*P* < 0.01, *r* = 0.85) higher compared with 2 h post exercise.

### The effect of exercise on corticosteroid hormones via the progesterone pathway

Corticosterone showed a significant main effect of time (F(2, 54) = 50.50, *P* < 0.001, ηp² =0.65) and a condition × time interaction (F(4, 54) = 3.32, *P* = 0.017, ηp² =0.20). Post hoc analysis indicated that only HIIE resulted in an increase in corticosterone from rest to post exercise (*P* < 0.001; Fig. 1a). Post exercise corticosterone levels following HIIE were higher (21.91 [25.58] ng·mL⁻¹, *P* = 0.005) than MICE (5.11 [10.09] ng·mL⁻¹) but were not different from RE (6.85 [6.56] ng·mL⁻¹; *P* = 0.008) following the adjusted post hoc threshold (*P* < 0.006). All exercise modes resulted in lower 2 h post exercise vs. post exercise concentrations (*P* < 0.001), with only MICE lower vs. resting concentrations (*P* < 0.01). MICE (*P* = 0.014, *r* = 0.76) and HIIE (*P* = 0.002, *r* = 0.89) significantly increased post exercise 11-dehydrocorticosterone concentrations compared with rest and at 2 h post exercise, 11-dehydrocorticosterone levels were significantly lower than resting (MICE: *P* < 0.05, *r* = 0.85; HIIE: *P* < 0.05, *r* = 0.65) and post exercise (MICE: *P* < 0.01, *r* = 0.85; HIIE: *P* < 0.01, *r* = 0.89) levels (Fig. 1b). RE did not change 11-dehydrocorticosterone levels throughout and there were no differences between groups at any time. Aldosterone showed a significant main effect of time (F(2, 54) = 98.78, *P* < 0.001, ηp² =0.79) and a condition × time interaction (F(4, 54) = 3.01, *P* = 0.026, ηp² =0.18). Post hoc analysis indicated that aldosterone increased post exercise across all conditions and was not different to resting levels at 2 h post exercise (Fig. 1c). Post exercise aldosterone concentrations following MICE (0.36 [0.23] ng·mL⁻¹; *P* < 0.01) and HIIE (0.45 [0.09] ng·mL⁻¹; *P* < 0.01) were higher than RE (0.23 [0.17] ng·mL⁻¹). 11-deoxycorticosterone was also measured and was undetectable in most participants. However, only HIIE resulted in 11-deoxycorticosterone elevations from undetectable levels to 0.03 (0.05) ng·mL⁻¹, which returned to resting at 2 h post exercise.

**Table 3:** The effect of exercise on body mass, blood pressure, heart rate, blood lactate and glucose.

	MICE			RE			HIIE			*P* value	
	Rest	Post	2 h Post	Rest	Post	2 h Post	Rest	Post	2 h Post	Time	Interaction
Body Mass (kg)	82.09 ± 11.90	81.87 ± 11.96	81.87 ± 11.93	82.37 ± 11.63	82.07 ± 11.78	81.93 ± 11.57	81.75 ± 11.83	81.49 ± 12.00	81.59 ± 11.99	0.02	0.801
Systolic blood pressure (mmHg)	119.00 (15.00)	144.00 (17.00)^**^	114.00 (8.00)^##^	116.00 (10.75)	128.00 (17.00)^aa^	117.00 (16.00)^##^	121.00 (10.00)	140.00 (10.00)^bb**^	116.00 (28.00)^##^	< 0.001	n/a
Diastolic blood pressure (mmHg)	67.00 (14.00)	68.00 (14.00)	60.00 (19.00)^##^	61.00 (11.00)	61.00 (13.00)^aa^	67.00 (29.00)	64.00 (23.00)	75.00 (19.00)^bb**^	66.00 (24.00)	0.023	n/a
Heart Rate (bpm)	60.00 (8.00)	104.00 (19.00)^**^	64.00 (10.00)^##^	57.00 (9.00)	110.00 (28.00)^**^	58.00 (8.00)^##^	58.00 (10.00)	110.00 (15.00)^**^	66.00 (15.00)^##^	< 0.001	n/a
Blood lactate (mmol.L)	0.76 (0.31)	2.15 (2.30)^***^	0.80 (0.45)^###^	0.63 (0.24)	6.15 (2.10)^aaa***^	1.06 (0.59)^**###^	0.60 (0.19)	5.30 (1.83)^aaa***^	0.91 (0.53)^###^	< 0.001	< 0.001
Blood glucose (mmol.L)	4.35 (0.40)	4.20 (0.50)	4.25 (0.28)	4.59 (0.24)	4.32 (0.17)^**^	4.13 (0.35)^**^	4.46 (0.20)	4.57 (0.69)^ab^	4.27 (0.48)^##^	< 0.001	n/a

Values are means ± SD or medians (IQR); *n* = 10 per group. Comparisons made between exercise modes by two-way mixed ANOVA or by Wilcoxon signed rank and Mann Whitney U tests. Holm-Bonferroni post hoc corrections were applied to all comparisons. ^aa^ denotes significantly (*P* < 0.01) different vs. MICE, ^aaa^ denotes significantly (*P* < 0.001) different vs. MICE, ^bb^ denotes significantly (*P* < 0.01) different vs. RE, ^**^denotes significantly (*P* < 0.01) different vs. rest, ^***^denotes significantly (*P* < 0.001) different vs. rest, ^##^denotes significantly (*P* < 0.01) different vs. post exercise, ^###^denotes significantly (*P* < 0.001) different vs. post exercise. HIIE, High-intensity intermittent exercise; MICE, moderate-intensity continuous exercise; RE, resistance exercise.

### The effect of exercise on corticosteroid hormones via the 17OH-progesterone pathway

Exercise had no effect on 21-deoxycortisol (Fig. 2a) however, 11-deoxycortisol showed a significant main effect of time (F(2, 54) = 89.81, *P* < 0.001, ηp² =0.77) and a condition × time interaction (F(4, 54) = 3.61, *P* = 0.011, ηp² =0.21). Post hoc analysis indicated post MICE, RE (both, *P* < 0.01) and HIIE (*P* < 0.001) increased compared with resting followed by lower levels at 2 h post exercise compared with post exercise (all, *P* < 0.001; Fig. 2c). Compared with resting, the 2 h post exercise levels were significantly lower following MICE (*P* = 0.008), RE and HIIE (both, *P* < 0.001). Cortisol showed a significant main effect of time (F(2, 54) = 49.26, *P* < 0.001, ηp² =0.65). Post hoc analysis indicated only HIIE significantly (*P* < 0.001) augmented post exercise cortisol levels compared with resting, however, all exercise conditions were lower at 2 h post exercise compared with post exercise (*P* < 0.001) and resting levels (*P* < 0.01; Fig. 2b). Cortisone showed a significant main effect of time (F(2, 54) = 23.69, *P* < 0.001, ηp² =0.47). Post hoc analysis indicated that MICE and RE did not (both, *P* > 0.05) increase post exercise cortisone levels compared with resting however, they were significantly (*P* < 0.01) lower at 2 r post exercise compared to post exercise concentrations. In contrast, HIIE increased post exercise cortisone compared with resting (*P* < 0.01) and returned to resting levels at 2 h post exercise (*P* < 0.01; Fig. 2d). There were no differences in 21-deoxycortisol, 11-deoxycortisol, cortisol and cortisone between exercise modes at any time.

### The effect of exercise on gonadal steroid hormones

MICE and RE did not significantly affect 17OH-progesterone levels. However, HIIE did observe significantly (*P* < 0.01) lower 2 h post exercise levels compared with post exercise (Fig. 3a). DHEA significantly increased post MICE, HIIE (both *P* < 0.01) and RE (*P* < 0.05) compared with resting and significantly (all, *P* < 0.01) decreased at 2 h post exercise compared with post exercise (Fig. 3e). Androstenedione showed a significant main effect of time (F(2, 54) = 78.13, *P* < 0.001, ηp² =0.74). Compared with resting post hoc analysis indicated increases post MICE (mean difference = 0.235 ng·mL⁻¹, 95% CI [0.076, 0.394], *P* = 0.005,), RE (mean difference = 0.172 ng·mL⁻¹, 95% CI [0.013, 0.330], *P* = 0.035) and HIIE (mean difference = 0.545 ng·mL⁻¹, 95% CI [0.386, 0.704], *P* < 0.001), followed by lower concentrations at 2 h post exercise compared with post exercise (all, *P* < 0.001) and resting (RE, *P* < 0.001; HIIE, *P* = 0.003; MICE, *P* = 0.035; Fig. 3b). A significant condition × time interaction was also observed (F(4, 54) = 5.69, *P* < 0.001, ηp² =0.30); however, post hoc analysis revealed no differences between exercise modes at any time. RE resulted in increased estrone concentrations at 2 h post exercise compared with post exercise (*P* = 0.008, *r* = 0.86), whereas HIIE increased estrone concentrations at 2 h post exercise compared with resting (*P* = 0.008, *r* = 0.83; Fig. 3f). Testosterone and 17b-estradiol showed significant main effects of time (testosterone: F(2, 54) = 5.69, *P* = 0.006, ηp² =0.17; 17b-estradiol: F(2, 54) = 3.30, *P* = 0.044, ηp² =0.09). However, post hoc analysis demonstrated no differences between timepoints within any exercise modality (Fig. 3c & g). Furthermore, exercise did not affect circulating 5a-DHT concentrations (Fig. 3d). Estriol was also measured but was undetected in all participants at all time points. Furthermore, no differences in 17OH-progesterone, testosterone, DHEA, estrone, 5a-DHT and 17b-estradiol were observed between exercise modes at any time.

### AUC analysis of steroid hormone responses to exercise

AUC analysis of steroid hormone responses to MICE, RE and HIIE are reported in Table 4. Significant differences between MICE, RE and HIIE were observed in the exposure time (ng/h) of corticosterone (*P* = 0.011) and aldosterone (*P* = 0.001). The rate of corticosterone production was significantly higher in HIIE (766.19 ± 487.18 ng/h) compared with MICE (342.16 ± 240.71 ng/h, *P* < 0.05) and RE (358.21 ± 172.51 ng/h, *P* < 0.05). Furthermore, compared with RE (9.80 ± 3.55 ng/h), MICE (15.52 ± 4.97 ng/h, *P* < 0.05) and HIIE (17.41 ± 3.60 ng/h, *P* < 0.01) significantly increased the rate of aldosterone exposure.

**Table 4:** AUC analysis of steroid hormone responses to MICE, RE and HIIE.

AUC (ng/h)				
Hormone	MICE	RE	HIIE	*P* value
Cortisol	8477.81 ± 2183.24	9644.18 ± 2003.45	10837.00 ± 2749.96	0.096
Cortisone	1889.97 ± 163.74	1935.56 ± 167.19	2004.37 ± 137.13	0.276
11-deoxycortisol	18.12 (14.39)	19.06 (8.37)	24.74 (16.25)	0.075
21-deoxycortisol	3.88 ± 1.88	3.69 ± 1.77	5.24 ± 2.77	0.244
Corticosterone	342.16 ± 240.71	358.21 ± 172.51	766.19 ± 487.18^aabb^	0.011
11-dehydrocorticosterone	79.76 ± 32.94	74.57 ± 28.79	114.71 ± 53.32	0.066
Aldosterone	15.52 ± 4.97	9.80 ± 3.55^aa^	17.41 ± 3.60^bbb^	0.001
17a-hydroxyprogesterone	73.44 (42.08)	79.57 (37.93)	75.38 (35.58)	0.380
Androstenedione	69.21 ± 18.02	72.92 ± 17.90	80.50 ± 20.99	0.413
Testosterone	430.80 ± 70.38	461.91 ± 71.59	472.11 ± 85.57	0.460
5a-DHT	35.93 ± 6.71	40.41 ± 16.00	37.77 ± 10.20	0.691
DHEA	280.00 (280.66)	376.63 (286.08)	423.94 (309.11)	0.154
Estrone	1.83 (0.86)	1.89 (0.72)	1.97 (0.70)	0.879
17b-Estradiol	1.87 ± 0.51	1.92 ± 0.41	2.02 ± 0.49	0.778

Values are means ± SD or medians (IQR); *n* = 10 per group. Comparisons made between exercise modes by one-way ANOVA or non-parametric Kruskal-Wallis tests with Holm-Bonferroni post hoc correction. ^aa^ denotes significantly (*P* < 0.01) different vs. MICE, ^aaa^ denotes significantly (*P* < 0.001) different vs. MICE, ^b^ denotes significantly (*P* < 0.017) different vs. RE, ^bb^ denotes significantly (*P* < 0.01) different vs. RE, ^bbb^ denotes significantly (*P* < 0.001) different vs. RE. 5-alpha-dihydrotestosterone, 5a-DHT; DHEA, Dehydroepiandrosterone; HIIE, High-intensity intermittent exercise; MICE, moderate-intensity continuous exercise; RE, resistance exercise.

### The effect of exercise on hormonal ratios

The ratio of testosterone to 17b-estradiol (T:E2), testosterone to cortisol (T:C), estrogens to progesterone (E:P) and androgens to estrogens (A:E) (Figure S2) can provide an indication of hormonal signaling and all showed significant (*P* < 0.001) main effects for time. Post hoc comparisons demonstrated that MICE did not change T:E2 from resting (249.02 [115.89]) to post exercise (268.48 [76.44], *P* = 1.00) to 2 h post exercise (206.19 [107.70], *P* = 0.557). However, RE significantly decreased T:E2 from resting (296.59 [117.32]) to post exercise (238.13 [46.75], *P* = 0.01, *r* = 0.79) and remained significantly lower at 2 h post exercise (234.67 [78.82], *P* = 0.006, *r* = 0.82). There were no significant differences between post exercise and 2 h post exercise T:E2 in MICE and RE conditions. HIIE did not change T:E2 from resting (260.15 [50.92]) to post exercise (234.98 [124.61], *P* = 0.432) but was significantly lower at 2 h post exercise (200.37 [85.63]) compared with resting (*P* = 0.014, *r* = 0.76) and post exercise (*P* = 0.02, *r* = 0.73).

T:C showed a significant main effect of time (F(2, 54) = 35.81, *P* < 0.001, ηp² =0.57). MICE did not change T:C from resting (0.06 ± 0.02) to post exercise (0.04 ± 0.02, *P* = 0.133) but was higher at 2 h post exercise (0.07 ± 0.04) compared with resting (mean difference = 0.022, 95% CI [0.008, 0.036], *P* = 0.003) and post exercise (mean difference = 0.029, 95% CI [0.017, 0.041], *P* < 0.001). Similarly, RE did not change T:C from resting (0.05 ± 0.01) to post exercise (0.05 ± 0.02, *P* = 0.573) but was higher at 2 h post exercise (0.06 ± 0.04) compared with resting (mean difference = 0.019, 95% CI [0.006, 0.033], *P* = 0.007) and post exercise (mean difference = 0.022, 95% CI [0.010, 0.034], *P* = 0.001). Following HIIE, T:C increased from resting (0.05 ± 0.03) to post exercise (0.04 ± 0.02, mean difference = 0.011, 95% CI [0.002, 0.020], *P* = 0.020) and was higher at 2 h post exercise (0.07 ± 0.05) compared with resting (mean difference = 0.020, 95% CI [0.006, 0.033], *P* = 0.007) and post exercise (mean difference = 0.031, 95% CI [0.018, 0.043], *P* < 0.001).

Compared with resting, MICE (0.05 [0.03]) E:P was not different at post exercise (0.05 [0.02], *P* = 0.625) or 2 h post exercise (0.07 [0.04], *P* = 0.105). Post exercise and 2 h post exercise were also not different (*P* = 0.084). Resting RE (0.05 [0.03]) E:P was not different to post exercise (0.05 [0.03], *P* = 0.492) 2 h post exercise (0.08 [0.04], *P* = 0.03). However, 2 h post exercise E:P was higher than post exercise (*P* = 0.01, *r* = 0.79). Resting HIIE (0.04 [0.03]) E:P was not different to post exercise (0.04 [0.02], *P* = 0.77) but increased at 2 h post exercise (0.07 [0.05], *P* = 0.004, *r* = 0.85), which was also higher than post exercise (*P* = 0.002, *r* = 0.89).

MICE did not change A:E from resting (256.66 [134.59]) to post exercise (277.30 [45.73], *P* = 0.846) to 2 h post exercise (174.35 [55.25], *P* = 0.064). There was also no difference (*P* = 0.064) between post exercise and 2 h post exercise A:E in MICE. Compared with resting RE (251.34 [138.96]), A:E was not significantly different at post exercise (305.97 [95.54], *P* = 0.131). However, 2 h post exercise (195.03 [73.86]) was lower compared with resting (*P* = 0.014, *r* = 0.76) and post exercise (*P* = 0.002, *r* = 0.89) in RE. HIIE resulted in an increase in A:E from resting (254.93 [67.47]) to post exercise (329.95 [193.19], *P* = 0.002, *r* = 0.89). A:E was lower at 2 h post exercise (161.88 [54.71]) compared with both resting and post exercise (both, *P* = 0.002, *r* = 0.89).

There were no significant differences between exercise conditions for any of the hormonal ratios.

## Discussion

The purpose of this study was to elucidate the effect of different exercise modalities on the steroid profile with a focus on acute responses under controlled conditions. To achieve this, we simultaneously profiled multiple steroid hormones within the same individuals using LC-MS/MS across three distinct exercise modalities: MICE, RE and HIIE, providing exploratory insights into acute endocrine responses in young active males. The major findings of this study are that (1) HIIE elicited the greatest metabolic disturbance and the largest increase in hormones related to energy mobilization, including cortisol, (2) All exercise resulted in increases in androgen precursors (DHEA, and androstenedione), consistent with transient endocrine activation, (3) HIIE uniquely increased 11-deoxycorticosterone, suggesting recruitment of a broader adrenal steroidogenic pathway via progesterone-derived corticosteroids and (4) RE and HIIE resulted in changes in estrogen-related markers (estrone, T:E2 and A:E) during recovery. Given the study design, sample size, and measurement constraints, findings should be interpreted as hypothesis-generating rather than definitive.

The current study demonstrates that compared with MICE, RE, and HIIE result in greater glycolytic demand as shown by the significantly higher increases in post-exercise lactate concentrations. Although peak lactate did not differ between RE and HIIE, lactate remained elevated 2 h after RE, consistent with previous research showing concentrations remain above baseline for > 60 min^8^, suggesting slower metabolic recovery. However, lactate concentrations at 2 h were not different between RE, HIIE, and MICE. Only HIIE resulted in significantly higher post exercise cortisol levels, and this elevation likely contributed to a parallel rise in cortisone concentrations. These findings are in line with previously reported studies where only exercise intensities > 60% V̇O_2max_ elicit significant increases in cortisol concentrations immediately post continuous exercise of 30 min^17,18^and HIIE^48^. Furthermore, cortisol decreased below baseline levels following 2 h post HIIE in accordance with previous findings^48^. Although the mechanisms underlying these responses were not assessed in the present study, previous observations indicate HIIE may acutely activate the hypothalamic-pituitary-adrenal axis, perhaps through suppressed glucocorticoid negative feedback^49^, thereby stimulating the release of adrenocorticotropic hormone^50^, promoting 11-deoxycortisol, cortisol, and cortisone synthesis^51^. These endocrine changes occurred alongside elevated heart rate and diastolic blood pressure post-HIIE, suggesting an association between neuroendocrine stress and cardiovascular strain rather than a causal relationship. It is generally accepted that cortisol increases lipolysis, gluconeogenesis, and inhibits peripheral glucose uptake^52^. Although these processes were not measured directly, blood glucose concentrations were significantly higher following HIIE compared with MICE and RE. However, the functional relevance of cortisol depends on its concentration, timing, and interaction with other hormones. Most in vivo studies on cortisol’s metabolic effects have relied on pharmacologically induced supraphysiological concentrations (300–850 ng·mL⁻¹)^53,54^, whereas the peak cortisol concentration observed in this study (285 ng·mL⁻¹) remained within a physiologically relevant range. Cortisol concentrations of at least 300 ng·mL⁻¹ appear to stimulate lactate-driven gluconeogenesis only in the presence of elevated glucagon after 3 h, with limited effect on blood glucose or glucose utilization before that time^55,56^. Regarding lipolysis, significant increases in circulating non-esterified fatty acids typically require co-infusion of high-dose cortisol and adrenaline^53^. Nevertheless, physiologically relevant cortisol levels (~ 145 ng·mL⁻¹) have shown to increase in vivo adipose transcription of lipolytic enzymes such as adipose triglyceride lipase and hormone-sensitive lipase^53^.

All exercise modes significantly increased post exercise DHEA and androstenedione concentrations however, testosterone and 5a-DHT concentrations remained unchanged. Previous research has investigated these hormonal responses to differing exercise modes independently using immunoassay techniques and is broadly consistent with our findings^12,13,47,57^. For example, high-load and low-load RE does not increase serum testosterone nor intramuscular 5a-DHT concentrations at 3 and 24 h post exercise^57^. Additionally, Tremblay et al. (2005) measured DHEA-sulfate and testosterone hourly for 4 h across rest, 40-, 80-, and 120-min runs, showing that DHEA-sulfate increased 20 min after 40 min of running, whereas testosterone rose during the first hour of ≥ 80 min runs and remained elevated 40 min post-exercise^47^. In contrast, maximal but not submaximal, aerobic exercise does increase serum testosterone and 5a-DHT concentrations until 60 min post exercise, indicating that exercise intensity and duration may be important determinants^12,13^. 5a-DHT is produced from the reduction of testosterone by 5α-reductase and has greater affinity for androgen receptors compared with testosterone, leading to genomic and non-genomic signaling^58,59^. However, supraphysiological doses of testosterone increases its affinity with androgen receptors^59^, increasing lean mass and strength in non-exercising males receiving 5α-reductase inhibitors, indicating that 5a-DHT is not essential for anabolic effects under such conditions^60^. The role of anabolic hormones for exercise induced adaptations remains unclear, as well-controlled studies have shown no association between exercise-induced changes in anabolic hormones and skeletal muscle hypertrophy^10,11^. Maintenance of the endocrine system may be a protective mechanism against metabolic disease and mortality. For example, males with higher physical activity levels over a 20-year period had higher baseline levels of testosterone, 5a-DHT and 17b-estradiol and a lower risk of metabolic syndrome and CVD-related death^61^. Furthermore, higher baseline levels of 5a-DHT up to 0.71 ng·mL⁻¹ are associated with reduced cardiometabolic risk and lower all-cause mortality^62^. Whether transient changes in post-exercise steroidogenic pathways contribute to endocrine maintenance in the face of age-related declines in sex hormones remains to be determined^63^.

Interestingly, both RE and HIIE augmented post-exercise estrone concentrations, although only HIIE showed values higher than resting levels. There is a lack of research on the effects of estrone in males due to its very low levels (0.03–0.04 ng·mL⁻¹) compared with females (~ 0.94 ng·mL⁻¹)^64^, and its physiological impact is also unclear, particularly as 17b-estradiol, the more metabolic active estrogen, did not change^65^. Although androstenedione increased across all exercise modalities, estrone increased only following RE and HIIE, suggesting precursor availability alone may not explain the response. The absence of changes in 17b-estradiol may reflect differences in the timing of the hormonal response relative to the sampling schedule. Estrogens primarily act upon their nuclear receptors – estrogen receptor (ESR) 1 and 2 – which are expressed in male skeletal muscle, adipose, bone, liver, and cardiovascular tissues^66^. Although the area is underexplored in males in response to exercise, ESR knockout mice exhibit dysfunctional glucose and lipid metabolism, impaired bone health, and compromised cardiac function^66^. Consequently, while the physiological relevance of the observed estrone responses with RE and HIIE remain uncertain, transient elevations in circulating estrogens warrant further investigation.

Furthermore, the balance of circulating androgens and estrogens may also provide insight into the regulation of homeostasis. Although, there were no differences in T:E2, E:P, A:E and T:C between exercise modes, RE and HIIE resulted in distinct shifts in hormonal balance. MICE had no effect on the T:E2, E:P, or A:E ratios at any time point. However, RE and HIIE differed in their immediate post exercise effects on T:E2 which decreased following RE only and A:E, which increased in response to HIIE only. In contrast, RE and HIIE both resulted in decreased T:E2 and A:E and increased E:P at 2 h post-exercise. These changes in hormonal ratios suggest that RE and HIIE elicited distinct shifts in the relative balance of circulating androgens and estrogens during recovery, whereas MICE did not. These observed changes may indicate a transient shift towards a more estrogenic hormonal profile during recovery and appear to be dependent on exercise intensity in males^12^. Additionally, all exercise modalities resulted in a higher T:C ratio at 2 h post-exercise, primarily due to lower cortisol concentrations. The increase in T:C at 2 h post-exercise may reflect a transient shift towards a less catabolic hormonal environment^2^. However, given the exploratory nature of these analyses and the low circulating estrogen concentrations in males, which may be more sensitive to analytical variability, the physiological relevance of these ratios remains uncertain and further work is required to determine whether these responses have functional significance.

Unsurprisingly, all exercise modalities perturbed fluid homeostasis as shown by significantly elevated aldosterone levels. Exercises that are continuous or of higher intensity lead to higher sweat rates and dehydration to regulate core temperature^67^, as confirmed by the higher aldosterone concentrations following MICE and HIIE. The greater relative intensity of HIIE also resulted in significantly greater elevations in the precursor to aldosterone, corticosterone, alongside elevated 11-dehydrocorticosterone levels, highlighting the mechanisms through which the body aims to increase fluid retention, maintain blood pressure, and sodium balance through reabsorption of fluids and electrolytes via the kidneys, salivary glands, sweat glands, and colon^4^. However, hydration status and plasma volume changes were not directly measured in this study, and therefore hormonal responses should be interpreted with caution, as they may partly reflect exercise-induced hemoconcentration rather than true alterations in hormone secretion^20^.

Previous research has demonstrated conflicting findings on the influence of exercise on endogenous hormone production^68^. These conflicting results are likely due variation in research designs, protocols, and reliance on ELISAs to determine hormonal concentrations. A major strength of the current study is that the same participants performed MICE, RE and HIIE in randomized order, at the same time of the day to control for internal biological and diurnal variations. Furthermore, all samples were simultaneously analyzed for 16 hormones via LC-MS/MS, allowing for greater precision, specificity, sensitivity, and accuracy compared with commonly used ELISAs^24^. Notwithstanding, there are some limitations to consider. An a priori sample size was not calculated, and the relatively small sample size alongside multiple outcome measures increases the risk of type I and II errors, therefore findings should be considered as exploratory and hypothesis-generating. Plasma volume was not measured and if this changed between exercise sessions and participants, it may have influenced hormonal concentrations without changes in hormonal synthesis, degradation or clearance. However, hormonal concentration changes due to shifts in plasma volume still regulate the interaction of hormones with target receptors, thus mediating physiologically relevant signal transduction. Furthermore, only hormonal plasma concentrations were measured and may not reflect changes within target tissues (e.g., muscle, adipose, bone), which mediate adaptations^57^. Although MICE and HIIE were matched for workload, RE was not matched for external work with aerobic protocols, as mechanical work is not directly comparable due to intermittent contractions, anaerobic contribution, eccentric loading, and differences in muscle mass. Therefore, the resistance protocol was designed to reflect standardized whole-body training consistent with ACSM recommendations rather than to equate external work. Participants were also provided with pre-testing instructions to reduce hormonal variations with lifestyle however, they were not recorded and may have impacted findings. Finally, the timing of sample collection and only 3 timepoints may not be optimal to capture peak hormone concentrations and AUC hormone exposure time. However, data demonstrated that most hormone levels at 2-h post exercise were similar to or below baseline, suggesting that peak concentrations likely occurred earlier – possibly at the immediate post-exercise point – but more frequent sampling would be required to confirm this.

To summarize, previous research has compared hormonal responses to exercise using parallel designs and analytical techniques that lack the precision to minimize biological variability, likely contributing to inconsistent results. This study simultaneously examined multiple hormonal responses to different exercise modalities using a randomized crossover design and LC-MS/MS, providing a controlled characterization of acute endocrine responses in young active males.

HIIE elicited greater acute metabolic and corticosteroid responses, while all exercise modalities increased androgen precursors. RE and HIIE showed changes in estrogen-related markers during recovery, whereas MICE showed comparatively smaller endocrine perturbations under the conditions studied.

These findings highlight mode-specific patterns of acute hormonal responses, suggesting that exercise intensity and metabolic demand – not duration alone – influence endocrine responses. However, given the exploratory nature of the study, small sample size, lack of plasma volume correction, and limited sampling timepoints, these findings should be interpreted with caution.

Future research should endeavor to elucidate the role of exercise-induced plasma hormonal responses utilizing LC-MS/MS, using more frequent sampling, while exploring their downstream effects on target tissues via genomic and non-genomic signaling in well-controlled acute and longitudinal studies, including investigations in females, older adults, and clinical populations, in whom hormonal regulation plays a central role in adaptation, health, and disease prevention.

## Supplementary Information

Below is the link to the electronic supplementary material.

Supplementary Material 1

## Data Availability

The datasets in the current study are available from the corresponding author on reasonable request, subject to ethical approval.
