# Supplementary material for: Steroid hormone responses to three exercise modalities assessed by liquid chromatography tandem mass spectrometry in a randomized crossover trial
Source: Sci Rep. 2026 Jun 29;16:20537. doi: 10.1038/s41598-026-58281-9 (PMC13332212; doi:10.1038/s41598-026-58281-9)
Supplement: Supplementary file 1 — Supplementary Material 1 [file 41598_2026_58281_MOESM1_ESM.docx]

**Table S1.** The CONSORT checklist.

|  | Section/topic | No | CONSORT 2025 checklist item description | Reported on page no. |
| --- | --- | --- | --- | --- |
|  | **Title and abstract** | | |  |
|  | Title and structured abstract | 1a | Identification as a randomised trial | 1 |
|  |  | 1b | Structured summary of the trial design, methods, results, and conclusions | 1 |
|  | **Open science** | | |  |
|  | Trial registration | 2 | Name of trial registry, identifying number (with URL) and date of registration | n/a |
|  | Protocol and statistical analysis plan | 3 | Where the trial protocol and statistical analysis plan can be accessed | n/a |
|  | Data sharing | 4 | Where and how the individual de-identified participant data (including data dictionary), statistical code and any other materials can be accessed | 21 |
|  | Funding and conflicts of interest | 5a | Sources of funding and other support (eg, supply of drugs), and role of funders in the design, conduct, analysis and reporting of the trial | 21 |
|  |  | 5b | Financial and other conflicts of interest of the manuscript authors | 21 |
|  | **Introduction** | | |  |
|  | Background and rationale | 6 | Scientific background and rationale | 2 |
|  | Objectives | 7 | Specific objectives related to benefits and harms | 2 |
|  | **Methods** | | |  |
|  | Patient and public involvement | 8 | Details of patient or public involvement in the design, conduct and reporting of the trial | n/a |
|  | Trial design | 9 | Description of trial design including type of trial (eg, parallel group, crossover), allocation ratio, and framework (eg, superiority, equivalence, non-inferiority, exploratory) | 4 |
|  | Changes to trial protocol | 10 | Important changes to the trial after it commenced including any outcomes or analyses that were not prespecified, with reason | n/a |
|  | Trial setting | 11 | Settings (eg, community, hospital) and locations (eg, countries, sites) where the trial was conducted | 4 |
|  | Eligibility criteria | 12a | Eligibility criteria for participants | 4 |
|  |  | 12b | If applicable, eligibility criteria for sites and for individuals delivering the interventions (eg, surgeons, physiotherapists) | 4 |
|  | Intervention and comparator | 13 | Intervention and comparator with sufficient details to allow replication. If relevant, where additional materials describing the intervention and comparator (eg, intervention manual) can be accessed | 4-5 |
|  | Outcomes | 14 | Prespecified primary and secondary outcomes, including the specific measurement variable (eg, systolic blood pressure), analysis metric (eg, change from baseline, final value, time to event), method of aggregation (eg, median, proportion), and time point for each outcome | 4-5 |
|  | Harms | 15 | How harms were defined and assessed (eg, systematically, non-systematically) | 4-5 |
|  | Sample size | 16a | How sample size was determined, including all assumptions supporting the sample size calculation | n/a |
|  |  | 16b | Explanation of any interim analyses and stopping guidelines | n/a |
|  | Randomisation: |  |  |  |
|  | Sequence generation | 17a | Who generated the random allocation sequence and the method used | 4 |
|  |  | 17b | Type of randomisation and details of any restriction (eg, stratification, blocking and block size) | 4 |
|  | Allocation concealment mechanism | 18 | Mechanism used to implement the random allocation sequence (eg, central computer/telephone; sequentially numbered, opaque, sealed containers), describing any steps to conceal the sequence until interventions were assigned | 4 |
|  | Implementation | 19 | Whether the personnel who enrolled and those who assigned participants to the interventions had access to the random allocation sequence | 4 |
|  | Blinding | 20a | Who was blinded after assignment to interventions (eg, participants, care providers, outcome assessors, data analysts) | n/a |
|  |  | 20b | If blinded, how blinding was achieved and description of the similarity of interventions | n/a |
|  | Statistical methods | 21a | Statistical methods used to compare groups for primary and secondary outcomes, including harms | 7 |
|  |  | 21b | Definition of who is included in each analysis (eg, all randomised participants), and in which group | 7 |
|  |  | 21c | How missing data were handled in the analysis | n/a |
|  |  | 21d | Methods for any additional analyses (eg, subgroup and sensitivity analyses), distinguishing prespecified from post hoc | n/a |
|  | **Results** | | |  |
|  | Participant flow, including flow diagram | 22a | For each group, the numbers of participants who were randomly assigned, received intended intervention, and were analysed for the primary outcome | 9 |
|  |  | 22b | For each group, losses and exclusions after randomisation, together with reasons | 9 |
|  | Recruitment | 23a | Dates defining the periods of recruitment and follow-up for outcomes of benefits and harms | 4 |
|  |  | 23b | If relevant, why the trial ended or was stopped | n/a |
|  | Intervention and comparator delivery | 24a | Intervention and comparator as they were actually administered (eg, where appropriate, who delivered the intervention/comparator, how participants adhered, whether they were delivered as intended (fidelity)) | 9 |
|  |  | 24b | Concomitant care received during the trial for each group | 9 |
|  | Baseline data | 25 | A table showing baseline demographic and clinical characteristics for each group | 9-10 + supp |
|  | Numbers analysed,  outcomes and estimation | 26 | For each primary and secondary outcome, by group:  ● the number of participants included in the analysis  ● the number of participants with available data at the outcome time point  ● result for each group, and the estimated effect size and its precision (such as 95% confidence interval)  ● for binary outcomes, presentation of both absolute and relative effect size | 9-16 |
|  | Harms | 27 | All harms or unintended events in each group | 9 |
|  | Ancillary analyses | 28 | Any other analyses performed, including subgroup and sensitivity analyses, distinguishing pre-specified from post hoc | n/a |
|  | **Discussion** | | |  |
|  | Interpretation | 29 | Interpretation consistent with results, balancing benefits and harms, and considering other relevant evidence | 18-20 |
|  | Limitations | 30 | Trial limitations, addressing sources of potential bias, imprecision, generalisability, and, if relevant, multiplicity of analyses | 20 |

Citation: Hopewell S, Chan AW, Collins GS, Hróbjartsson A, Moher D, Schulz KF, et al. CONSORT 2025 Statement: updated guideline for reporting randomised trials. BMJ. 2025; 388:e081123. <https://dx.doi.org/10.1136/bmj-2024-081123>
© 2025 Hopewell et al. This is an Open Access article distributed under the terms of the Creative Commons Attribution License (<https://creativecommons.org/licenses/by/4.0/>), which permits unrestricted use, distribution, and reproduction in any medium, provided the original work is properly cited.

*We strongly recommend reading this statement in conjunction with the CONSORT 2025 Explanation and Elaboration and/or the CONSORT 2025 Expanded Checklist for important clarifications on all the items. We also recommend reading relevant CONSORT extensions. See [www.consort-spirit.org](http://www.consort-spirit.org).

## Liquid-chromatography mass spectrometry for targeted steroid analysis

Sixteen (16) steroids were measured in the plasma samples by extraction and then LC-MS/MS analysis. Plasma, calibration standards (16 points, ranging from 0.0025 – 500 ng), quality control (QC) samples and blanks were dispensed manually as aliquots (150 µL) into individual wells of a 2mL 96 deep-well polypropylene plate (Waters, UK). An internal standard solution of all isotopically labelled standards (2,3,4-[^13^C_3_]-androstenedione, 2,3,4-[^13^C_3_]-testosterone, 2,3,4-[^13^C_3_]-5alpha-dihydrotestosterone, 2,2,3,4,4-[^2^H]_5_-dehydroepiandrosterone, 9,11,12,12-[^2^H]_4_-cortisol, (2,2,4,6,6,9,12,12-[^2^H]_8_-cortisone, 2,2,4,6,6,17a,21,21-[^2^H]_8_-corticosterone, (2,2,4,6,6-[^2^H]_5_-11-deoxycortisol, (2,2,4,6,6,21,21-[^2^H]_8_-21-deoxycortisol 2,3,4-[^13^C_3_]-17beta-estradiol, 2,3,4-[^13^C_3_]-estrone, 2,2,4,6,6,21,21,21 -[^2^H]_8_-aldosterone and (2,2,4,6,6,21,21,21 -[^2^H]_8_ -17alpha-hydroxyprogesterone in methanol was added (20 µL; various ng) to each well except for the double blanks. The plate was agitated on a plate shaker (2 mins) and transferred to a Biotage^®^ Extrahera^™^ automated sample processor (Biotage, Uppsala, Sweden) where formic acid (250 µL, 0.1% v/v) was added to each well. Samples were incubated at room temperature (18‑22 ^°^C; 5 mins) before being transferred to the SLE+ 400 plate by the robot. Once on the SLE+ plate the samples were loaded onto the SLE material under positive pressure using compressed air. Following a wait (5 min) the analytes were eluted from the SLE material into a deep-well collection plate by positive pressure following the addition of dichloromethane/propan-2-ol (98:2; 4x 450 µL). The eluate was dried down under a stream of heated oxygen free nitrogen (OFN, 40 °C) on an SPE Dry^TM^ Dual Sample Concentrator System (Biotage, Uppsala, Sweden). Once dry the extracts were dissolved in water/methanol (70:30, 70 µL), the plate was sealed then shaken on a plate shaker (10 mins) before injecting (20 µL) directly from the 96-well plate for liquid chromatography tandem mass spectrometry (LC-MS/MS) analysis.

LC-MS/MS analysis was performed using a I-Class UPLC (Waters, UK) interfaced to a QTRAP 6500+ (AB Sciex, Warrington, UK) mass spectrometer. Instrument control and data acquisition were achieved using Sciex Analyst^®^ 1.6.3 Software. Data were integrated and analysed using software package Sciex MultiQuant 3.0.3. Steroids were separated on a Kinetex C18 (150 x 2.1 mm; 2.6 um) liquid chromatography system using water and methanol with ammonium fluoride as a mobile phase system at 0.3 mL/min as described in Denham et al. [47].

The mass spectrometer was operated in positive/negative ion polarity switching in electrospray ionisation (ESI) mode using a TurboIonSpray source and data were collected in unit resolution (0.7 *m/z* full width at half maximum). The TurboIonSpray source was operated at 600^o^C with a voltage of +5.5 kV and -4.5 kV, a Curtain Gas of 30 psi, nitrogen nebuliser ion source gas 1 (GS1) and heater ion source gas 2 (GS2) of 40 psi and 60 psi, respectively. Compound specific parameters were optimised for multiple reaction monitoring (MRM) transitions by infusing 100 ng/mL or 10 ng/mL of each steroid standard solution into the source. The curtain, source, exhaust and CAD gas was delivered using an MS Table 1N Nitrogen and dry air generator Table (Peak Scientific, Scotland, UK). Two transitions were selected for each steroid – the quantitative and qualitative ion - and their optimised declustering potential, collision exit potential and collision energy are listed in Table 1 and Table 2.

| **Table S2.** Mass Spectrometry parameters for positive ion multiple reaction monitoring on QTrap 6500+ mass spectrometer | | | | | | | |
| --- | --- | --- | --- | --- | --- | --- | --- |
| **ID** | **Q1 Mass (Da)** | **Q3 Mass (Da)** | **DP**  **(volts)** | **CE**  **(volts)** | **CXP**  **(volts)** | **Time**  **(min)** |  |
| Cortisol 1 | 363.1 | 121.2 | 76 | 31 | 8 | 3.4 |  |
| Cortisol 2 | 363.1 | 91.0 | 76 | 83 | 10 | 3.4 |  |
| Cortisone 1 | 361.1 | 163.1 | 81 | 31 | 26 | 2.9 |  |
| Cortisone 2 | 361.1 | 77.0 | 81 | 107 | 10 | 2.9 |  |
| Corticosterone 1 | 347.1 | 121.1 | 76 | 29 | 8 | 5.3 |  |
| Corticosterone 2 | 347.1 | 90.9 | 76 | 75 | 12 | 5.3 |  |
| 11-Dehydrocorticosterone 1 | 345.1 | 121.2 | 66 | 31 | 12 | 3.6 |  |
| 11-Dehydrocorticosterone 2 | 345.1 | 91.2 | 66 | 83 | 40 | 3.6 |  |
| 11-Deoxycortisol 1 | 347.0 | 97.0 | 71 | 27 | 12 | 5.7 |  |
| 11-Deoxycortisol 2 | 347.0 | 109.0 | 71 | 33 | 16 | 5.7 |  |
| 11-Deoxycorticosterone 1 | 331.2 | 97.0 | 86 | 29 | 16 | 7.5 |  |
| 11-Deoxycortisterone 2 | 331.2 | 109.0 | 86 | 31 | 12 | 7.5 |  |
| Testosterone 1 | 289.1 | 97.0 | 101 | 29 | 12 | 7.6 |  |
| Testosterone 2 | 289.1 | 109.2 | 101 | 31 | 6 | 7.6 |  |
| Androstenedione 1 | 287.0 | 97.0 | 61 | 27 | 14 | 6.9 |  |
| Androstenedione 2 | 287.0 | 78.9 | 61 | 67 | 10 | 6.9 |  |
| 5a -Dihydrotestosterone 1 | 291.3 | 255.2 | 116 | 21 | 30 | 8.9 |  |
| 5a-Dihydrotestosterone 2 | 291.3 | 91.0 | 116 | 55 | 10 | 8.9 |  |
| Dehydroepiandrosterone 1 | 271.1 | 235.1 | 106 | 17 | 12 | 8.0 |  |
| Dehydroepiandrosterone 2 | 271.1 | 188.1 | 106 | 17 | 12 | 8.0 |  |
| 17OH-Progesterone 1 | 331.0 | 109.1 | 66 | 31 | 12 | 8.1 |  |
| 17OH-Progesterone 2 | 331.0 | 96.9 | 66 | 29 | 12 | 8.1 |  |
| D4-Cortisol | 367.3 | 121.1 | 80 | 29 | 16 | 3.3 |  |
| D8-Cortisone | 369.2 | 169.0 | 96 | 33 | 20 | 2.8 |  |
| D8-Corticosterone | 355.3 | 125.1 | 56 | 31 | 8 | 5.2 |  |
| D5-11-Deoxycortisol | 352.1 | 100.1 | 101 | 29 | 12 | 5.6 |  |
| D8-21-Deoxycortisol | 355.2 | 319.1 | 51 | 23 | 20 | 5.1 |  |
| ^13^C_3_-Testosterone | 292.1 | 100.0 | 96 | 29 | 12 | 7.6 |  |
| ^13^C_3_-Androstenedione | 290.2 | 100.1 | 31 | 27 | 12 | 6.8 |  |
| ^13^C_3_-5a-Dihydrotestosterone | 294.2 | 258.3 | 61 | 21 | 12 | 8.9 |  |
| D8-17OH-Progesterone | 339.2 | 100.1 | 111 | 29 | 12 | 8.0 |  |
| D5-Dehydroepiandrosterone | 294.1 | 258.2 | 21 | 13 | 28 | 7.9 |  |

| **Table S3.** Mass spectrometry parameters for negative ion multiple reaction monitoring on Qtrap 6500+ mass spectrometer | | | | | | | |
| --- | --- | --- | --- | --- | --- | --- | --- |
| **Steroid** | **Q1 Mass (Da)** | **Q3 MASS**  **(Da)** | **DP**  **(volts)** | **CE**  **(volts)** | **CXP**  **(volts)** | **Time**  **(min)** |  |
| Aldosterone 1 | 359.1 | 188.9 | -70 | -24 | -21 | 2.62 |  |
| Aldosterone 2 | 359.1 | 331.0 | -70 | -22 | -35 | 2.62 |  |
| D8-Aldosterone | 367.2 | 193.9 | -70 | -24 | -21 | 2.59 |  |
| Estrone (1) | 269.1 | 144.9 | -150 | -48 | -15 | 7.20 |  |
| Estrone (2) | 269.1 | 142.9 | -150 | -70 | -15 | 7.20 |  |
| ^13^C_3_-Estrone | 272.1 | 147.8 | -150 | -52 | -21 | 7.20 |  |
| Estradiol (1) | 271.0 | 144.9 | -110 | -52 | -21 | 7.00 |  |
| Estradiol (2) | 271.0 | 182.9 | -110 | -52 | -19 | 7.00 |  |
| ^13^C_3_-Estradiol | 274.0 | 147.9 | -110 | -48 | -29 | 7.00 |  |
| Estriol (1) | 287.1 | 171.0 | -110 | -52 | -21 | 2.54 |  |
| Estriol (2) | 287.1 | 145.0 | -110 | -52 | -19 | 2.54 |  |
| ^13^C_3_-Estriol | 290.2 | 173.9 | -110 | -48 | -29 | 2.55 |  |

Multi-steroid data collected by the LC-MS/MS method was evaluated using a purpose-built quantitation method using MultiQuant 3.0.3 with MRM transitions and retention times (RT) of steroids and isotopically labelled internal standards (IS) steroids defined, according to the mass transitions of the quantitative and qualitative ions and analytical standard retention times of the chromatographic method described in Table 1 and Table 2. The quantitation method integrated all peaks at the defined retention times, resulting in a peak area for each IS and steroid detected in each calibration standard point, and each biological sample. Linear regression of the peak area ratio of the steroid to IS against the calibration standard amount. Linear regression was used to calculate the amount of steroid in each biological sample.

**Table S4.** Inter-assay accuracy (%RME) and precision (%RSD) for each steroid following automated supported liquid extraction and LC-MS/MS analysis.

| Range | A | 11DOC | E3 | 11S | 21DF | Aldo | DHT | E2 | E1 |
| --- | --- | --- | --- | --- | --- | --- | --- | --- | --- |
| LLOQ (ng/ml) | 0.125 | 0.125 | 0.0125 | 0.0625 | 0.1875 | 0.0625 | 1.25 | 0.0625 | 0.25 |
| Inter-assay precision (%RSD) | 17.5 | 7.8 | 19.0 | 18.6 | 17.8 | 13.0 | 8.3 | 8.6 | 15.9 |
| Inter-assay accuracy (%RME) | 9 | −2 | 9 | 7.5 | −3 | 0 | 0 | 3 | 5 |
| ULOQ (ng/ml) | 2.5 | 2.5 | 2.5 | 2.5 | 2.5 | 2.5 | 2.5 | 2.5 | 2.5 |
| Inter-assay precision (%RSD) | 5.7 | 4.2 | 3.2 | 2.4 | 6.7 | 10.6 | 8.2 | 3.1 | 7.1 |
| Inter-assay accuracy (%RME) | −4.7 | 1.0 | 2.8 | 2.1 | 1.3 | 0.8 | 3.0 | 3.0 | 5.8 |
|  |  |  |  |  |  |  |  |  |  |
| Range | **A4** | **T** | **E** | **B** | **P4** | **DHEA** |  |  |  |
| LLOQ (ng/ml) | 0.1 | 0.5 | 0.25 | 0.25 | 0.25 | 1.25 |  |  |  |
| Inter-assay precision (%RSD) | 10.0 | 10.7 | 2.4 | 15.0 | 11.1 | 18.2 |  |  |  |
| Inter-assay accuracy (%RME) | 11 | −4 | −1 | −12 | 3 | 2 |  |  |  |
| ULOQ (ng/ml) | 10 | 10 | 25 | 25 | 5 | 25 |  |  |  |
| Inter-assay precision (%RSD) | 5.7 | 5.1 | 2.6 | 4.0 | 2.4 | 3.7 |  |  |  |
| Inter-assay accuracy (%RME) | 1.1 | −1.7 | 0.4 | 0.2 | 3.8 | 1.8 |  |  |  |
|  |  |  |  |  |  |  |  |  |  |
| Range | **P5** | **17OHP4** | **F** |  |  |  |  |  |  |
| LLOQ (ng/ml) | 1 | 0.5 | 5 |  |  |  |  |  |  |
| Inter-assay precision (%RSD) | 14.4 | 12.5 | 10.4 |  |  |  |  |  |  |
| Inter-assay accuracy (%RME) | −10 | −3 | 2 |  |  |  |  |  |  |
| ULOQ (ng/ml) | 5 | 5 | 500 |  |  |  |  |  |  |
| Inter-assay precision (%RSD) | 13.6 | 7.6 | 8.0 |  |  |  |  |  |  |
| Inter-assay accuracy (%RME) | −0.3 | −1.2 | 1.9 |  |  |  |  |  |  |

**Table S5.** Inter-assay accuracy and precision (*n* = 6) of quality control serum at low, medium and high levels, defined in the table, for each QC serum panel following automated supported liquid extraction and LC-MS/MS analysis.

| Analyte | Range (ng/ml) | Low QC level (ng/ml) | Acc (%) | RSD (%) | Medium QC level (ng/ml) | Acc (%) | RSD (%) | High QC level (ng/ml) | Acc (%) | RSD (%) |
| --- | --- | --- | --- | --- | --- | --- | --- | --- | --- | --- |
| T | 0.05–10 | 0.189 | 98.6 | 14.3 | 1.51 | 96.8 | 4.5 | 8.02 | 101.3 | 9.5 |
| A4 | 0.05–10 | 0.291 | 92.4 | 18.9 | 1.14 | 104.4 | 5.6 | 9.45 | 104.3 | 9.9 |
| ALDO | 0.0125–5 | 0.098 | 99.8 | 16.3 | 0.223 | 110.7 | 15.8 | 0.932 | 107.8 | 5.3 |
| F | 2.5–500 | 24.90 | 105.0 | 14.8 | 59.80 | 107.2 | 19.1 | 171.0 | 110.9 | 14.0 |
| E | 0.125–25 | 2.010 | 108.8 | 16.3 | 12.00 | 109.8 | 14.9 | 29.00 | 106.7 | 11.0 |
| B | 0.125–25 | 0.868 | 101.2 | 7.9 | 4.360 | 102.7 | 18.6 | 30.10 | 110.3 | 13.0 |
| 11DOC | 0.0125–2.5 | 0.076 | 100.4 | 9.3 | 0.197 | 94.2 | 11.7 | 0.984 | 101.1 | 14.7 |
| S | 0.0125–2.5 | 0.320 | 98.5 | 16.7 | 1.500 | 92.7 | 13.1 | 9.850 | 108.4 | 7.2 |
| P4 | 0.025–5 | 0.281 | 86.2 | 16.9 | 3.080 | 94.0 | 6.9 | 15.30 | 101.0 | 9.0 |
| 17OHP4 | 0.025–5 | 0.296 | 112.0 | 11.3 | 1.470 | 109.9 | 11.6 | 8.860 | 114.7 | 13.8 |
| E2 | 0.0125–2.5 | 0.081 | 94.9 | 7.1 | 0.414 | 104.1 | 6.3 | 2.610 | 102.9 | 6.3 |
| 21DF | 0.0125–2.5 | 0.090 | 107.8 | 11.4 | 0.380 | 113.9 | 14.8 | 2.290 | 111.2 | 9.8 |
| DHT | 0.0125–2.5 | 0.080 | 107.6 | 0.2 | 0.374 | 96.8 | 10.9 | 1.130 | 112.8 | 5.5 |
| DHEA | 0.125–25 | 1.930 | 108.0 | 5.5 | 11.70 | 106.8 | 11.0 | 18.20 | 107.5 | 9.9 |

Enrolment

Assessed for eligibility (n= 10)

Excluded (n=0)

Randomised (n= 10)

Allocated in random sequence

Allocated to MICE or HIIE or RE (n= 10)

Received allocated intervention (n= 10)

Allocated to MICE or HIIE or RE (n= 10)

Received allocated intervention (n= 10)

Allocated to MICE or HIIE or RE (n= 10)

Received allocated intervention (n= 10)

Analysis

Analysed for primary outcome (n= 10)

Excluded from analysis (give reasons) (n= 0)

**Fig S1** CONSORT 2025 flow diagram of the progress through the phases of a randomised trial of two groups (that is, enrolment, intervention allocation, follow-up, and data analysis). HIIE, High-intensity intermittent exercise; MICE, moderate-intensity continuous exercise; RE, resistance exercise

| Table S6. Physical activity, cardiopulmonary exercise test and maximal strength testing characteristics. | |
| --- | --- |
| *Characteristic* | *Value* |
| ***Physical activity*** | |
| Walking (MET-min/week) | 2681.0 ± 2565.2 |
| Moderate (MET-min/week) | 4655.5 ± 3255.5 |
| Vigorous (MET-min/week) | 1744.0 ± 1106.0 |
| Total (MET-min/week) | 6342.5 ± 3604.7 |
| ***Cardiopulmonary exercise test*** | |
| V̇O_2peak_ (ml**^.^**kg**^.^**min^-1^) | 47.5 ± 6.7 |
| V̇O_2peak_ (L**^.^**min^-1^) | 3.9 ± 0.3 |
| V̇O_2peak_ (% predicted) | 115.7 ± 11.7 |
| Maximal work rate (Watts) | 347.3 ± 33.3 |
| Maximal work rate (% predicted) | 116.1 ± 14.1 |
| Heart rate peak (b**^.^**min^-1^) | 186.6 ± 8.9 |
| Peak oxygen pulse (ml**^.^**beat^-1^) | 20.8 ± 2.3 |
| VO_2_ at VT1 (ml**^.^**kg**^.^**min^-1^) | 26.9 ± 5.6 |
| VO_2_ at VT1 (L**^.^**min^-1^) | 2.2 ± 0.4 |
| Work rate at VT1 (Watts) | 347.3 ± 33.3 |
| Heart rate at VT1 (b**^.^**min^-1^) | 130.3 ± 15.0 |
| ***Maximal strength testing (1RM)*** | |
| Seated leg press (kg) | 254.9 ± 60.8 |
| Chest press (kg) | 91.8 ± 33.9 |
| Leg extension (kg) | 127.9 ± 35.7 |
| Lat pull down (kg) | 82.1 ± 15.6 |
| Upright row (kg) | 38.6 ± 9.6 |
| Shoulder press (kg) | 70.5 ± 26.4 |
| Values are expressed as means ± SD, n=10. *MET,* metabolic equivalent; *V̇O2peak_,_* peak oxygen consumption; *VT1,* first ventilatory threshold, *1RM,* one repetition maximum strength | |


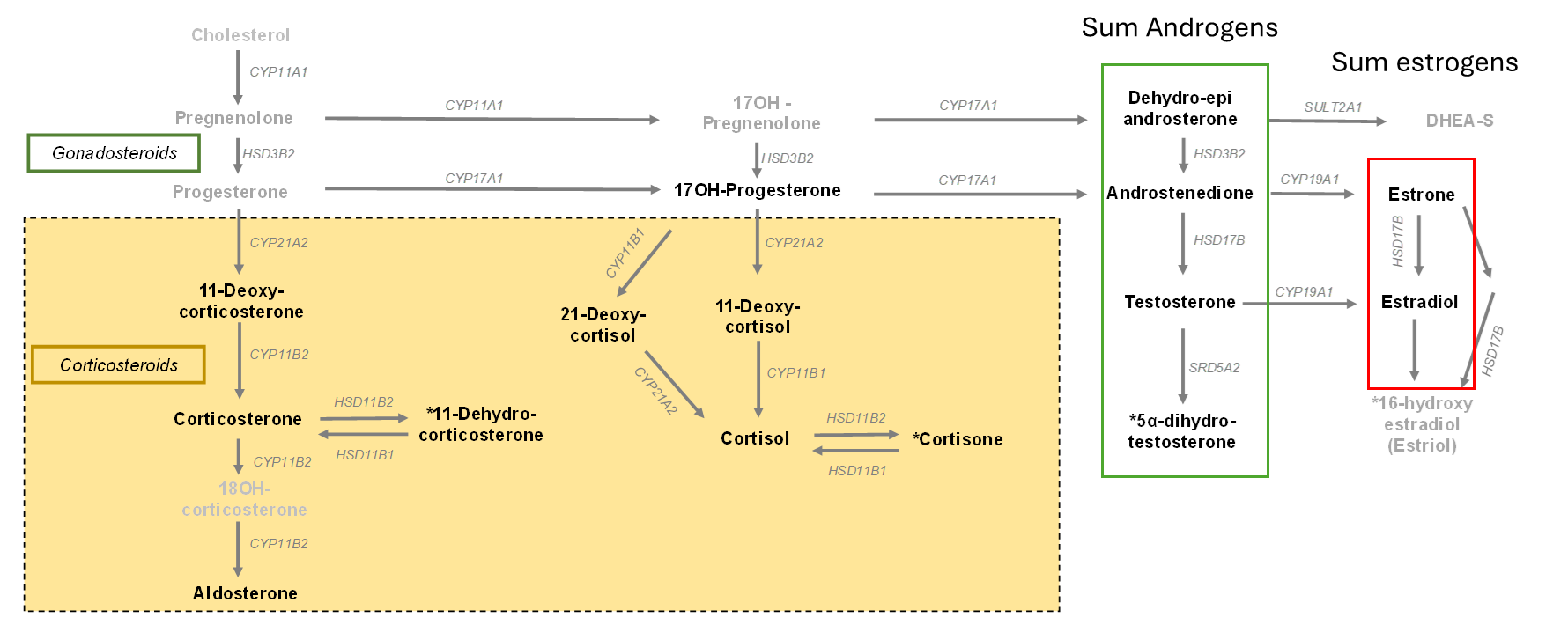


**Figure S2:** Illustration of the conceptual framework underpinning the hormone ratios. The testosterone:cortisol (T:C) ratio reflects anabolic–catabolic balance, while testosterone:estradiol (T:E2) represents key bioactive androgen and estrogen balance. The androgens:estrogens (A:E) ratio was calculated as the sum of circulating androgens relative to sum estrogens, providing an index of overall androgenic–estrogenic balance. The estradiol:progesterone (E:P) ratio reflects downstream steroid metabolism. These ratios were interpreted within the steroidogenic pathway to assess exercise-induced shifts in hormonal balance rather than isolated hormone concentrations.
